# Presenting a conceptual framework for an HIV prevention and care continuum and assessing the feasibility of empirical measurement in Estonia: A case study

**DOI:** 10.1371/journal.pone.0240224

**Published:** 2020-10-09

**Authors:** Anneli Uusküla, Peter Vickerman, Mait Raag, Josephine Walker, Dimitrios Paraskevis, Ksenia Eritsyan, Vana Sypsa, Dmitry Lioznov, Radko Avi, Don Des Jarlais

**Affiliations:** 1 Department of Family Medicine and Public Health, University of Tartu, Tartu, Estonia; 2 Population Health Sciences, Bristol Medical School, University of Bristol, Bristol, United Kingdom; 3 Medical School, National and Kapodistrian University of Athens, Athens, Greece; 4 National Research University Higher School of Economics, Saint-Petersburg, Russian Federation; 5 Smorodintsev Research Institute of Influenza, St. Petersburg, Russia; 6 First Pavlov State Medical University, St. Petersburg, Russia; 7 Department of Microbiology, University of Tartu, Tartu, Estonia; 8 College of Global Public Health, New York University, New York, NY, United States of America; FHI360, UNITED STATES

## Abstract

**Objective:**

We aim to show the feasibility of using an integrated prevention and care continuum (PCC) model as a complete and improved tool for HIV control measurement and programming. Alignment of prevention and care continua is essential to further improve health outcomes and minimize HIV transmission risk.

**Design:**

Cross-sectional study.

**Methods:**

Data from 977 persons who inject drugs (PWID) collected in 2011–2016 in Tallinn, Estonia, were used to construct an HIV PCC for PWID, stratified by risk for acquiring or transmitting HIV infection and by coverage of combined interventions. We also estimated the average protective effect of current levels of intervention provision.

**Results:**

74.4%, 20.3% and 35.2% of PWID were currently using needle and syringe programmes (NSP), drug treatment and HIV testing, respectively. 51.1% of current PWID were HIV seropositive and of those 62.5% were currently on ART and 19.0% were virally suppressed. Across the PCC, individuals moved between categories of being aware and ever using drug treatment (resulting in -50% “leakage”); from ever having used to currently using drug treatment (-59%); between “ever testing” and “current (continuous) testing” (-62%); and from self-reported antiretroviral therapy (ART) adherence to viral suppression (-70%). Use of prevention services was higher among those at risk of transmission (HIV positive). The overall reduction in acquisition risk among HIV-negative PWID was 77.7% (95% CrI 67.8–84.5%), estimated by the modelled protective effects of current levels of NSP, drug treatment and ART compared to none of these services.

**Conclusions:**

Our findings suggest that developing a cohesive model for HIV prevention and treatment is feasible and reflects the bi-directional relationships between prevention and care. The integrated continuum model indicates the major factors which may predict the epidemic course and control response.

## Introduction

The construct of ‘care continua’ is used to evaluate the effectiveness of care and treatment for chronic and infectious disease [1]. The Joint United Nations Programme on HIV and AIDS (UNAIDS) goal of “90% diagnosed, 90% on antiretroviral therapy (ART) and 90% virologically suppressed” represents a reduced, operational continuum of care [2]. In parallel to the care continuum, HIV prevention continua have also been proposed [3–5]. Here the focus shifts from infected individuals to those at risk and who could benefit from primary prevention interventions (i.e. from pre-exposure prophylaxis (PrEP) [6] or combined interventions to block sexual acquisition of HIV [3, 7]). Horn et al (2016) advance the concept and propose an integrated primary and secondary HIV prevention continuum [8].

To further improve health outcomes and minimize transmission risk among those who are infected with HIV, alignment of both prevention and care continua is essential. However, there are few models/applications that illustrate the link between primary prevention and the care (secondary prevention) continuum.

### Conceptual framework for a Prevention and Care Continuum (PCC)

Public health prevention comprises primary (pre-event), secondary (event) and tertiary interventions (post-event). Primary HIV prevention reduces the incidence of transmission, whereas secondary HIV prevention focuses on early detection and prompt ART treatment, and tertiary prevention promotes quality of life and prolongs longevity through ART, cancer treatment, and addressing opportunistic infections.

**Fig 1:**
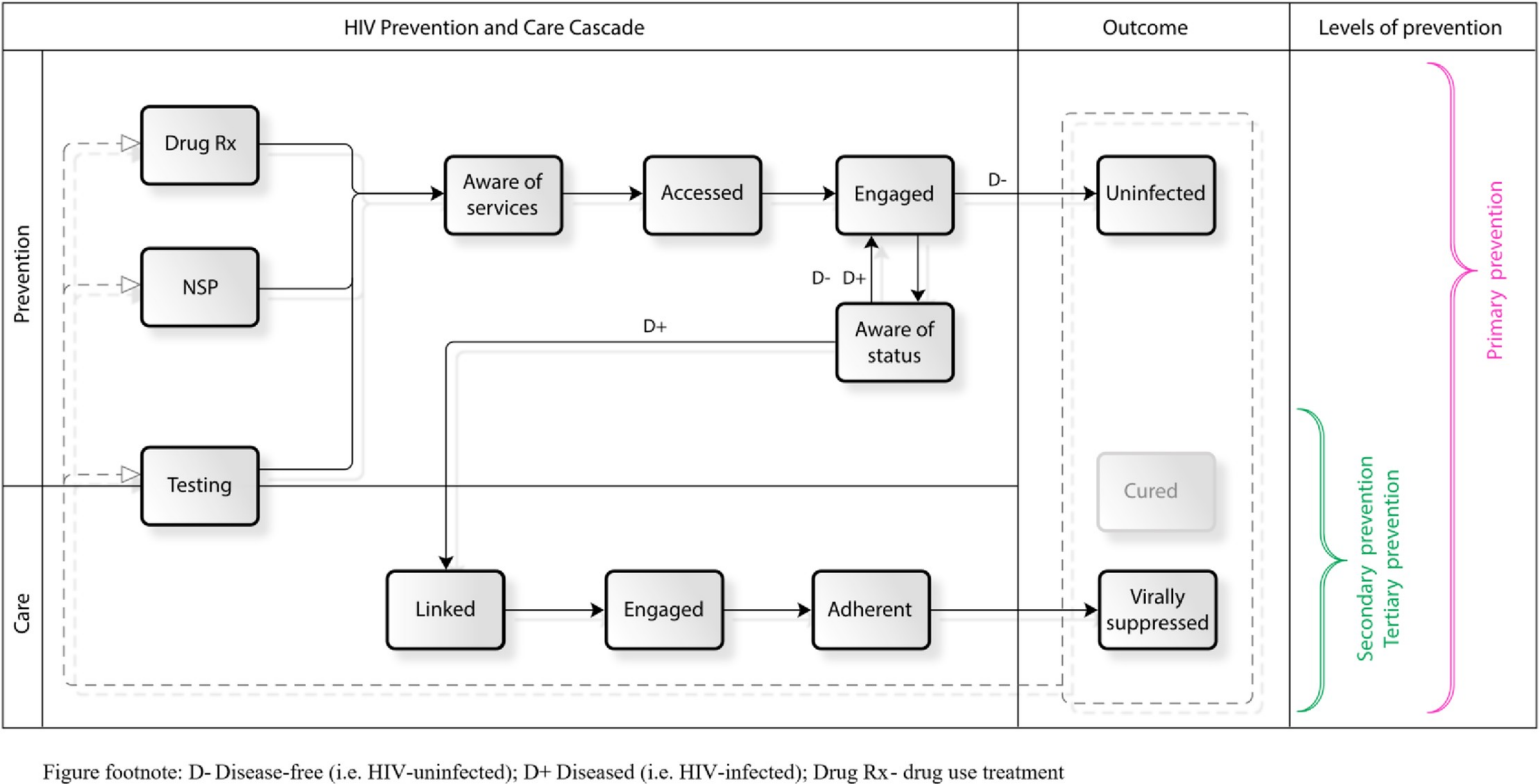
Conceptual framework for the Prevention and Care Continuum (PCC): An example of HIV infection among PWID.

We propose a continuum that employs preventive interventions (primary prevention) among the key population, and continues with secondary and tertiary prevention interventions that incorporate testing, treatment and engagement in care. All primary prevention services and HIV testing are potential entry points in the PCC, as they provide contact with the service / healthcare delivery systems for the members of the key population. Testing is a critical component in the joint continuum both as a metric to continuously assess the effectiveness of the primary HIV prevention cycle and as an opportunity to streamline successful linkage with the HIV care continuum (Fig 1). By providing HIV testing, the population can be categorised into those who are at-risk of acquiring (HIV negative) and those transmitting (HIV positive) infection. For those who are HIV negative, the continuum entails cyclical engagement in preventive interventions and repeated testing. For those who are HIV positive, the process involved engagement in the care continuum (provision of ART, achieving virologic suppression, and maintaining chronic care).

We outline a new model of an integrated HIV prevention and care continuum, and test its application on data collected from persons who inject drugs (PWID) in Tallinn, Estonia, from 2011 to 2016. The endpoint measure is an estimate of the average protective effect of current care models compared to no interventions.

## Methods

### Setting

Although HIV infection is preventable, significant HIV transmission continues across the WHO European region at an estimated rate of 16 newly diagnosed infections per 100 000 population in 2018 [9]. The rates and overall numbers of people diagnosed with HIV are highest in the East of the region (45 per 100 000 population), where injection drug use and unprotected sexual contacts between PWID and their non-PWID sex partners is a significant driver of the HIV epidemic [9].

In 2018, Estonia reported the third highest rate of new HIV diagnoses (14 per 100 000) after Latvia (17 per 100 000) and Malta (15 per 100 000) in the European Union and the European Economic Area (EU/EEA) [9]. Despite relatively high use of needle and syringe programmes (NSP) and antiretroviral therapy (ART) [9] among PWID, Estonia has a sustained high HIV sero-prevalence (> 50%) epidemic with a moderate incidence among PWID. Prevention services (NSP, drug treatment, voluntary counselling and testing) are available, and have probably contributed to reducing the HIV incidence in Tallinn [10, 11] but the volume and intensity of services may still be suboptimal [12, 13]. Most PWID in Estonia have accurate knowledge about the transmission and prevention of HIV [14]. PrEP for PWID was not available in Estonia before 2019.

#### Preventive interventions for people who inject drugs (PWID)

A comprehensive package of preventive interventions for PWID [15] includes needle and syringe programmes (NSP), drug treatment programmes (including medication-assisted treatment, MAT), HIV testing, and treatment with antiretroviral therapy (ART). Some interventions, such as MAT for substance abuse act as primary prevention (by reducing unsafe injection practices) [16, 17] and secondary (care) intervention (improving ART uptake, adherence and retention) [18]. Likewise, ART serves a dual role: 1) as community level primary prevention by reducing the population viral load, and thereby the likelihood of exposure, as well as at an individual level via pre-exposure prophylaxis (PrEP) and antepartum ART; and 2) treatment as care [19].

### Design

Using data from cross-sectional surveys undertaken among PWID in Estonia, we constructed an HIV prevention and care continuum for PWID, stratified by the risk of acquiring or transmitting HIV.

### Data

Data for the current analysis were collected from three cross-sectional studies conducted biennially from 2011 to 2016 in Tallinn using standardized methods of subject recruitment and of behavioral and biological data collection. Detailed descriptions of the studies and methods have been published elsewhere [10, 13]. These studies used respondent-driven sampling (RDS) [20] to recruit current PWID. Potential participants were eligible for the study if they: lived in Tallinn or Harju County; were at least 18 years of age; spoke Estonian or Russian; reported having injected in the previous two months; were able and willing to provide informed consent; and agreed to donate a blood sample for HIV testing. RDS recruitment began with the non-random selection of ‘seeds’ (n = 6 in 2011, 2013 and n = 8 in 2016) purposefully selected to represent diverse PWID types (by age, gender, ethnicity, main type of drug used, and HIV status). After they had participated in the study, subjects were provided with coupons for recruiting up to three of their peers (other PWIDs). Coupons were uniquely coded to link participants to their survey responses and to biological specimens, and for monitoring who recruited whom. Participants who completed the study received a primary incentive (a 10 Euro grocery store voucher) for participation in the study and a secondary incentive (a 5 Euro grocery store voucher) for each peer recruited (peers had to attend, be eligible and complete study procedures for the recruiter to receive the incentive). Data from participants was collected in private, via face-to-face interviewer-administered structured questionnaire lasting approximately 30–45 minutes. The questionnaire contained multiple-choice answer options and rating scales based on the WHO Drug Injecting Study Phase II survey [21]. Questions elicited information on PWID demographics, injection and other drug use, sexual risk behaviour, HIV testing and treatment history and the use of various HIV/harm reduction-related services. Data were collected from the Tallinn West Central Hospital Infectious Diseases clinic (the sole HIV care provider in this region) using standardized forms, including dates of visits/tests and viral load values.

Venous blood was collected and tested for the presence of HIV antibodies using commercially available test kits [ADVIA Centaur CHIV Ag/Ab Combo (Siemens Healthcare Diagnostics, Inc., Erlangen, Germany)]. Participants received pre- and post-test HIV counselling.

### Statistical analysis

Data from the questionnaires were used to create operational definitions for the nine stages of the care continuum (Table 1); the definition of each stage was derived or adapted from evidence informed standards [22, 23]. For this demonstration analysis, we selected four priority interventions (NSP, HIV testing, drug treatment, ART) based on the target group and local availability of the services. For each intervention we recorded participant awareness, access, and self-reported continuous use. We also provided estimates of the PCC steps for combined interventions. We stratified the prevention component of the PCC by the risk of acquiring (HIV-negative PWID) or transmitting (HIV-positive PWID) the infection.

**Table 1:** Indicator list of metrics for monitoring the PCC.

*For all participants*:
• Step 1. Awareness of services: ‘Are you aware of the… [NSP, drug treatment, HIV testing, ART] services?’ (a separate question for each service).
• Step 2. Accessed services: ‘Have you ever used the… [NSP, drug treatment, HIV testing, ART] services?’ (a separate question for each service).
• Step 3. Currently using services: ‘Have you used the NSP services during the last 6 months?’, ‘When did you have your last HIV test? (having had a HIV test within the last 6 months was defined as current use) [22], and: ‘Are you currently receiving treatment to modify your drug use? (drug treatment)’
• Step 4. Awareness of HIV-infected status: ‘What was the result of your last HIV test?’ (for those ever tested); those who had never tested were considered as not aware.
*For those HIV-infected*:
• Step 5. Linkage with care: ‘Have you ever visited the HIV clinic for care?’, or, ‘Have you ever been on ART?’
• Step 6. Engaged (retained) in care: ‘Have you visited the HIV clinic in the last 6 months?’ [23]
• Step 7. Currently on ART: ‘Are you currently taking ART?’
• Step 8. Adherence to ART: we used a visual analogue scale (VAS) representing the percentage of medication taken relative to that which had been prescribed [24]. Participants were asked to mark the point on a line ranging from 0% to 100% that represented ‘How much of your HIV medication have you taken?’ Optimal adherence (> = 80%) and suboptimal (<80%) adherence were defined [25].
• Step 9. Viral suppression: we estimated the proportion of study subjects with viral load (HIV-RNA) below <400 copies/mL [26] based on clinic data (a viral load estimate obtained within 90 days of the study visit) (in 2013) or on participant self-reporting of the latest viral load reading (in 2016).

**Fig 2:**
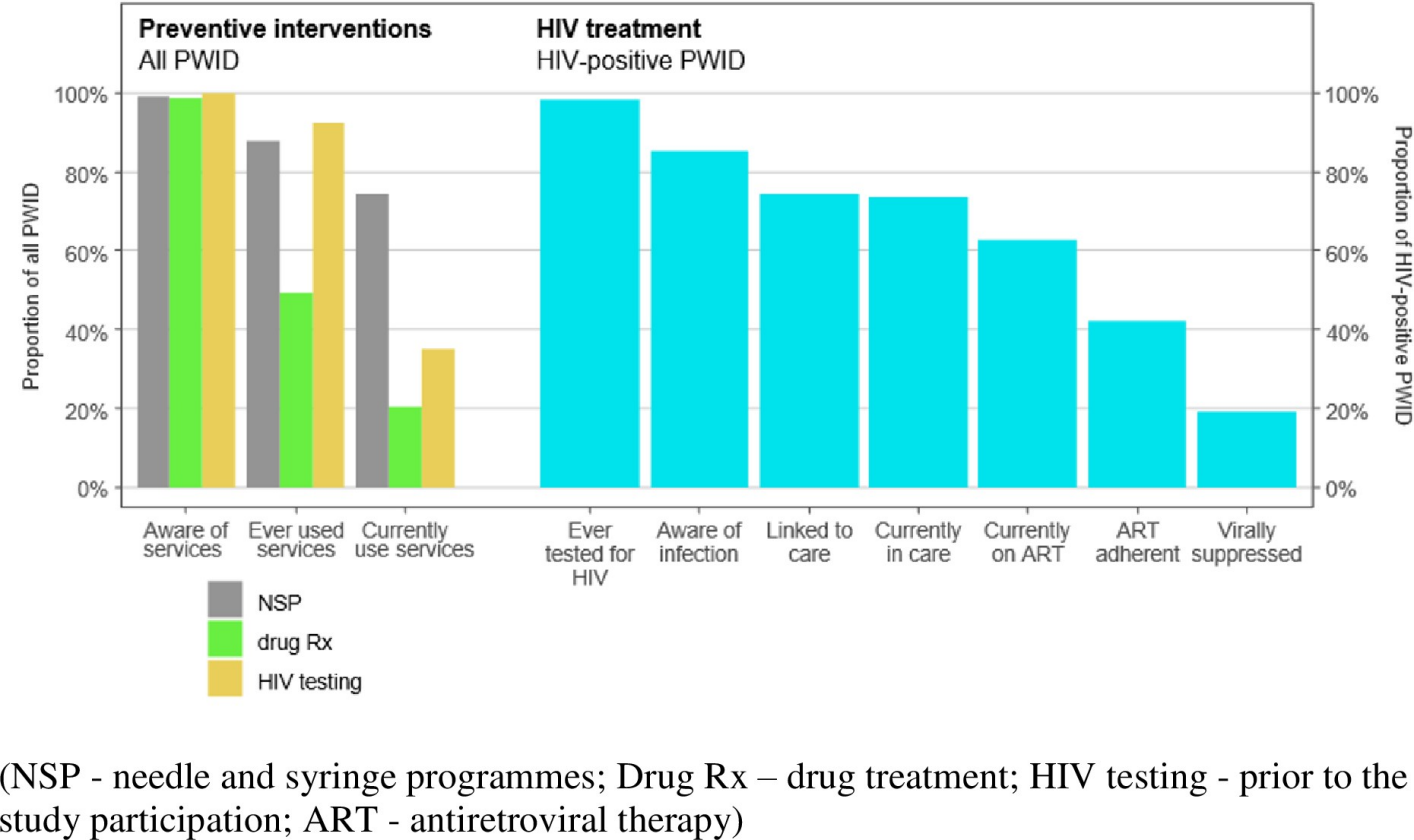
HIV prevention and care continuum for people who inject drugs in Tallinn, Estonia (2011–2016).

**Fig 3:**
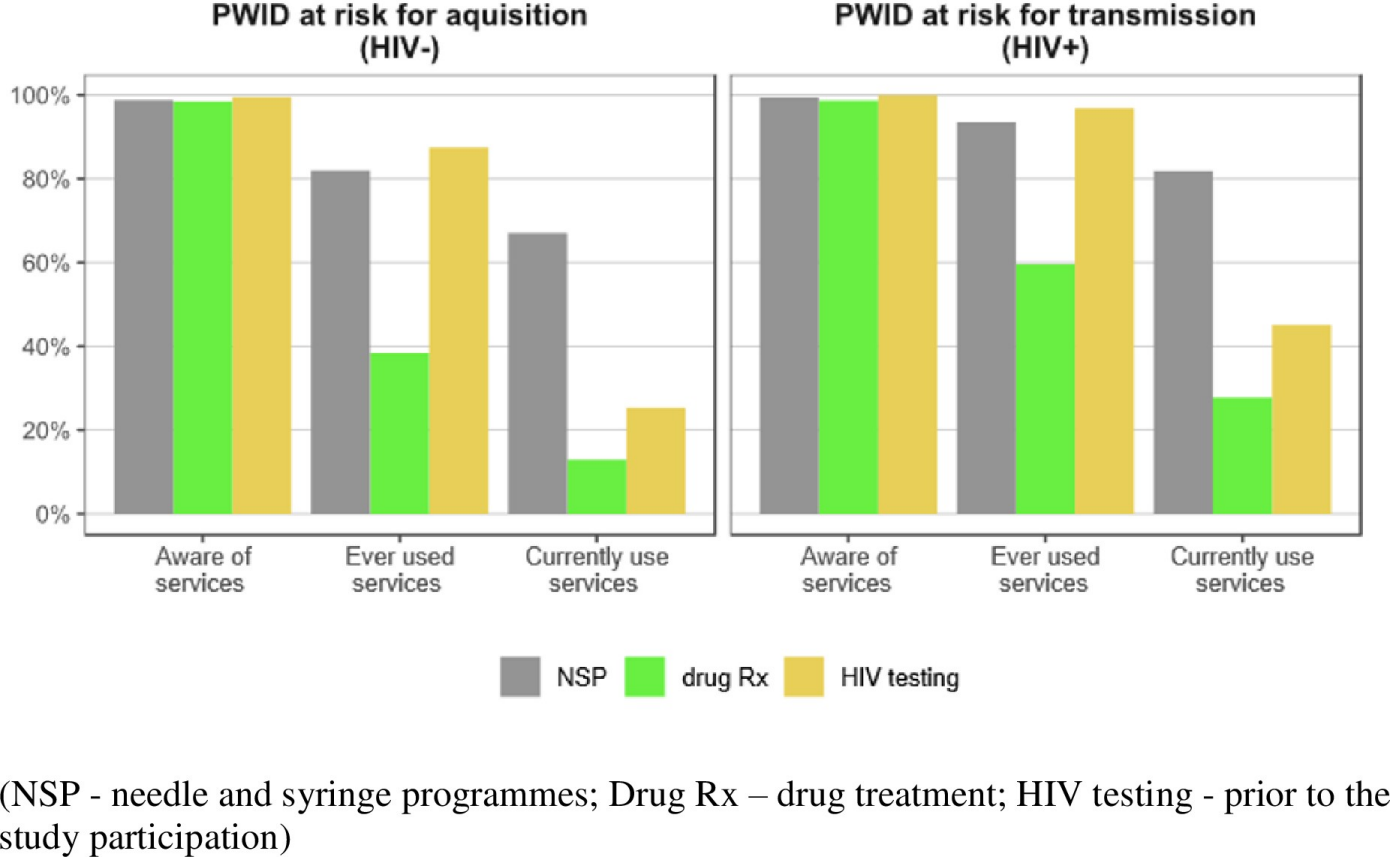
Reported use of HIV prevention interventions among HIV positive and HIV negative people who inject drugs in Tallinn, Estonia (2011–2016) (HIV+ n = 529; HIV- n = 448).

To summarise data on all PWID enrolled during the period, and to estimate continuum proportions (Figs 2 and 3), we used statistical environment R [27] with the package RDS [28]. The recruitment data (i.e., the number of potential participants who the respondent knew within the target population and the coupon numbers of each respondent and his/her recruiter) were used to derive RDS sequential sampling (SS) estimates with 95% confidence intervals (CIs) of the continuum proportions of interest. As people move along the continuum, there is a loss to follow up, and this "leakage" creates a retention cascade. We used those proportions to describe PWID at each step (the number of participants who responded ‘Yes’ to the current step indicator question (= numerator) divided by the total or all in the strata (= denominator). To quantify leakage occurring between continuum steps, relative differences were calculated. Details of the proportions of PWID comprising each step observed are given in S1 Data (S1 and S2 Tables in S1 Data).

We also used data from the PCC on the proportion of HIV-negative and positive PWID at different stages of the prevention and treatment continuum to estimate the possible degree to which the susceptibility and infectivity in each group may have decreased in this population due to all prevention interventions. For this, we used best available estimates for the efficacy of MAT (54%, 95% CI 33–68%) and NSP (58%; 95% CI 19–78%) based on two systematic reviews [29, 30], as well as viral load data from PWID on ART to estimate the prevention effectiveness of ART [31]. For instance, to estimate the average decrease in susceptibility, the proportion of HIV-negative PWID on just MAT (2.7%) or just NSP (55.5%) were multiplied by the efficacy of MAT (m) and NSP (n), respectively, and the proportion on both MAT and NSP (13.2%) was multiplied by (1-(1-m)(1-n)), which assumes MAT and NSP act independently when in combination. This is in line with evidence on the combined efficacy of MAT and NSP (76%) for reducing HCV transmission as found in a recent Cochrane review [17]. The average decrease in infectivity for HIV-positive PWID was estimated similarly, but also included the effect of ART. This assumed that all HIV-positive PWID with suppressed viral load (57%) or with unsuppressed viral load had a decrease in infectivity related to the log_10_ difference in viral load compared to HIV-infected PWID not on ART. No data were available on the viral load of PWID not on ART in this study, so we assumed a viral load for European PWID not on ART from a previous study [31, 32] (4.79 log_10_ copies/ml, IQR 4.11–5.27), and a factor difference in HIV transmission risk of 2.45 (95% CI 1.85–3.26) per log_10_ increment of viral load [33]. This suggested that the prevention efficacy of ART with suppressed or unsuppressed viral load was 97% (95% CI 91–99%) and 51% (95% CI 3.7–79%), respectively. Uncertainty in our average efficacy estimates was quantified through undertaking 1000 random draws from the uncertainty bounds of each intervention’s estimated efficacy, and 1000 samples with replacement of the proportion of the population in each harm reduction category (on NSP, MAT, none or both for each of HIV-positive PWID on or off ART, or HIV-negative PWID). We also estimated the overall protective efficacy by combining the reduction in susceptibility among HIV-negative PWID (a) and reduction in infectivity of HIV-positive PWID (t) as 1-(1-a)(1-t). In all of these calculations, we estimated the average proportion of the protective effect (together with the 95% credibility interval, CrI) due to each intervention by considering the relative protection provided by each intervention on its own.

Ethical approval for the studies was obtained from the Ethics Review Board of the University of Tartu, Estonia in 2011, 2013 and 2016. Written informed consent was secured from all participants.

## Results

### Characteristics of the study population

The analysis contains data on 977 current PWID. Participants had a mean age of 31.7 years, and 77.9% were men. On average, they reported starting to inject at the age of 20 (mean 19.7) years, and close to a quarter (22.8%) reported injecting at least once daily. The majority (72.0%) injected mainly opioids. Both receptive and distributive sharing was reported over the previous 6 months. The former was defined as acquiring used syringes or needles to use for personal injections (23.9%); the latter defined as giving, lending, renting or selling syringes or needles that the individual had already used to someone else to inject (24.3%). About half (51.1%, 95% CI 43.6–58.5) of the current PWID were HIV-seropositive, and of those infected, 14.8% were unaware of their HIV positive status. Of those who were HIV positive and aware, 62.5% and 73.3%, respectively were on ART.

Given self-reported evidence of negative HIV tests within the preceding 6 months (n = 161) and HIV seropositivity at study recruitment (n = 12), 6.1% (95% CI 3.9–8.4%) were recently infected.

### Prevention and care continuum

In Fig 2, we describe the HIV prevention and care continuum among PWID beginning from the awareness of prevention/care services up to the outcomes (the proportions of those who were HIV negative, and HIV positive virally suppressed). Awareness of prevention and harm reduction services is extremely high among PWID. Most had been tested for HIV (92.2%), used NSP (87.9%), and approximately half (49.2%) had used drug treatment at some point. As expected, current use of these services was reported at somewhat lower levels for each service. Of those reporting current drug treatment, 87% were on MAT. Of those who were HIV positive, 85.3% were aware of their serostatus, almost three-quarters (74.5%) had accessed HIV treatment; 73.4% were engaged in HIV care; 62.5% were currently on ART; and 19.0% were virally suppressed.

Use of prevention services differed by HIV serostatus (Figs 2 and 3; S1, S2 Tables in S1 Data). Specifically, among the HIV positive, 96.9% had received HIV testing vs 87.5% who were seronegative, *p* < 0.001; similarly, NSP was 93.5% vs 82.0%, *p <* 0.001; and drug treatment 59.6% vs 38.4%, *p* < 0.001, for seropositive and seronegative PWIDs respectively (Fig 3).

Across the PCC, substantial leakage occurred as individuals moved between categories of “being aware of” and “ever using” drug treatment (relative decreases: among all participants 50%; among HIV_neg_ 61%; and among HIV_pos_ 40%) and from “ever having received” to “currently receiving” drug treatment (among all participants 59%; among HIV_neg_ 66%; among HIV_pos_ 53%). Substantial leakage occurs between “ever been (HIV) tested” and “current (continuous) testing” (among all participants 62%; among HIV_neg_ 62%) steps of the continuum.

### Prevention and care continuum—combined interventions

Most HIV-negative PWID (78.1%) were currently engaged in one or more of the applicable prevention interventions (HIV testing in the last 6 months, drug treatment, NSP): 44.4% reported using a single intervention (37.2% NSP, 1.4% drug treatment, 5.8% HIV testing); 25.2% participated in 2 interventions; and 4.0% were currently engaged in all three interventions (4.5% of participants missed one or more of these questions).

Less than one-tenth (7.6%) of HIV-positive PWID were not current users of NSP, and were neither receiving drug treatment nor ART, whereas 16.4% were currently receiving all three interventions. Slightly over one-third (35.1%) reported using a single intervention (28.0% NSP, 0.3% drug treatment, 6.5% ART) and 40.0% participated in 2 interventions.

It should be noted that 86% of those currently on drug treatment also reported NSP use (indicative of continuous injecting while on drug treatment). However, current injecting was an eligibility criterion for the studies, so that people receiving drug treatment who were not currently injecting would not have been included.

**Fig 4:**
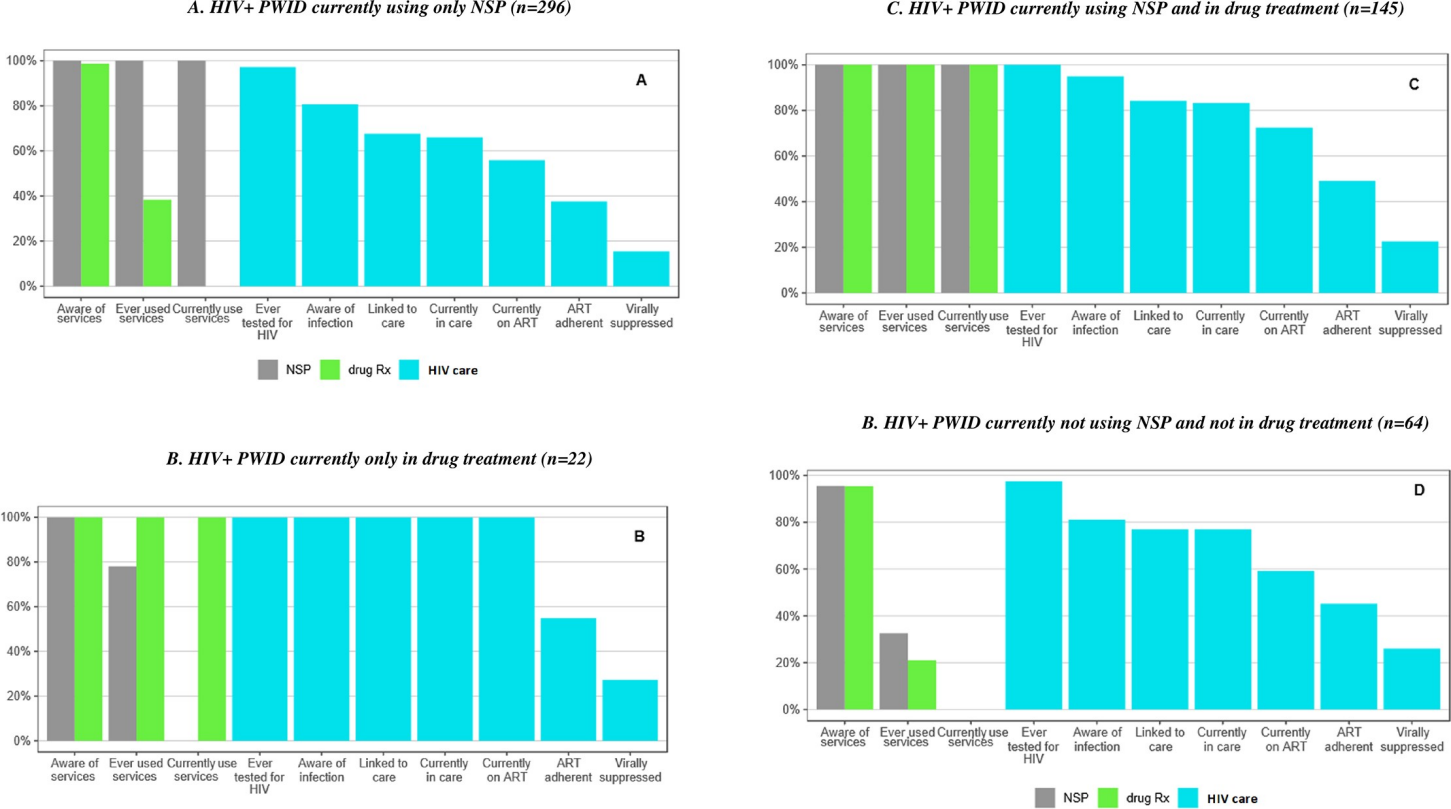
HIV prevention and care continua for combined interventions among HIV+ people who inject drugs in Tallinn, Estonia (2011–2016). (4A - HIV+ PWID currently using only NSP; 4B - HIV+ PWID currently only in drug treatment, 4C - HIV+ PWID currently using NSP and in drug treatment, 4D - HIV+ PWID currently not using NSP and not in drug treatment).

There were significant differences in ART coverage between HIV seropositive participants associated with their use of combined preventive interventions (p < 0.001, Fig 4). Those most likely to receive ART were the HIV seropositive who were receiving treatment for their addiction, but this group was small at less than 4% of the seropositive subgroup. Nearly 1 in 4 participants were receiving both NSP and drug treatment; of these, 73% were on ART.

**Fig 5:**
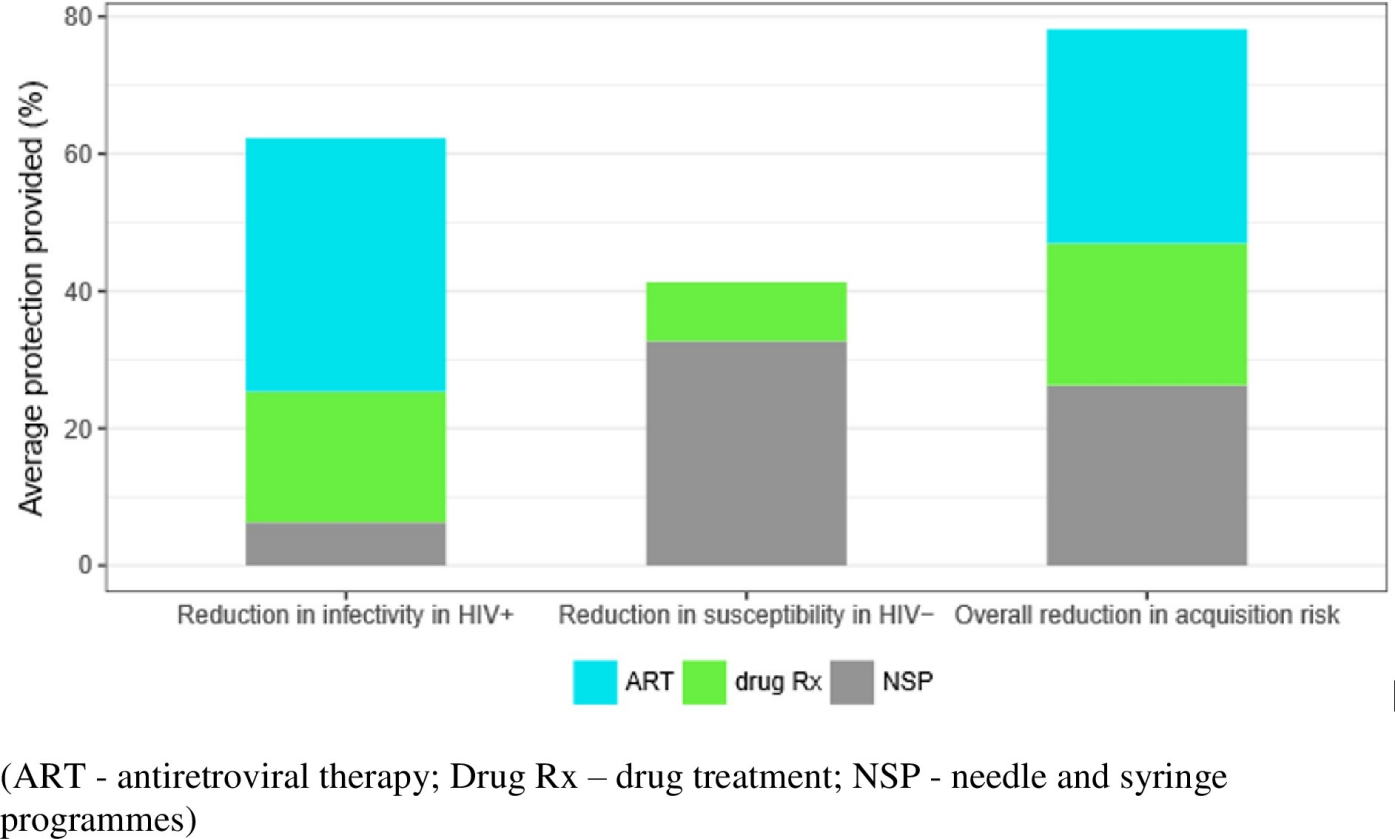
Relative decrease in infectivity and susceptibility among HIV+ and HIV- persons who inject drugs (PWID), and resulting overall reduction in acquisition risk due to the existing coverage of MAT, NSP and ART in PWID in Tallinn (median of 1000 runs).

Our modelling results (Fig 5) suggest that the average reduction in susceptibility in HIV-negative PWID due to all prevention interventions is 41.0% (95% CrI 24.5–54.5%), while the average reduction in the likelihood of HIV-positive PWID transmitting the virus is 62.3% (95% CrI 52.8–70.3%). These protective effects produce an overall reduction in the risk of acquiring HIV infection among PWID of 77.7% (95% CrI 67.8–84.5%) compared to the risk in the absence of NSP, drug treatment and ART. Most of this protective effect is due to the provision of ART to those already infected (31.0%; 95% CrI 23.7–36.7%) and of NSP (25.8%; 95% CrI 14.6–34.3%) whereas treatment for drug addiction provides a smaller proportion (20.9%; 95% CrI 14.6–28.5%) due to its lower coverage (Fig 5). Most of the uncertainty in these protection estimates is due to uncertainty in the proportion of people on NSP and MAT and the effectiveness of NSP and ART.

## Discussion

The construct of a joint prevention and care continuum provides an integrated framework for evaluating complex interactions between prevention and care. The added value in our integrated continuum derives from 1) having more detail in identifying potential weak points across the prevention-care continuum; 2) identifying interactions between weaknesses in prevention and in care; and 3) offering the ability to consider bi-directional relationships in care and prevention which can achieve maximum protection from interventions.

We distinguish two important sub-populations of PWID: those at risk of acquiring and those at risk of transmitting HIV. Significant differences manifest in preventive intervention coverage between these sub-groups. Monitoring the proportion of people living with HIV who are on successful ART is useful for optimizing individual clinical outcomes and control, but does not support program managers in understanding the effectiveness of prevention efforts among the currently uninfected at-risk. This is especially critical among PWID due to the potential for a small number of HIV positive individuals to initiate an outbreak.

We observed a high HIV incidence among current PWID in Tallinn, which suggests a failure of primary prevention interventions. The low coverage with combined prevention–strongly promoted by international health authorities [34]–is apparent from the joint continuum model. Importantly, low levels of drug treatment coverage among these (mainly opioid-injecting) PWID extends across the continuum. This lack of drug treatment probably affects the ART treatment outcome: low ART adherence is a cross-cutting challenge affecting PWIDs and is associated with an unacceptably low proportion of HIV positive PWID achieving viral suppression (19%). Being able to take into account bi-directional relationships in care and prevention can help to inform/prioritize responses to achieve maximum protection from interventions.

We can speculate that the high population level use of NSP (contributing 26% of overall reduction in risk) in Estonia ameliorates some of the deficiencies of other services. Our model indicates that drug treatment and NSP in the HIV-negative decreases susceptibility to HIV acquisition by 41.3%, while among seropositive PWID, combined drug treatment, NSP and ART decreases infectivity by 61.4%. Combining these protective effects yields an overall 77.7% reduction in the acquisition risk among HIV-negative PWID. Nevertheless, additional research must be devised to understand the degree to which components act synergistically, and to determine which factors have the greatest single capacity to effect control, at the individual and population levels.

A focus on HIV surveillance data on new diagnoses among PWID is needed to understand prevention gaps and missed prevention opportunities, with rapid translation to “reverse engineer” [8] primary HIV prevention priorities.

### Limitations

We are aware of the predictive limitations of cross-sectional studies. Here, we are not using these cross-sectional data to prove causation between successive stages in the integrated continuum. It is important to note that the steps in the prevention and care continuums denote causal pathways, and that these causal pathways have been demonstrated in previous research. Some of the causal pathways are simple “necessary but not sufficient” conditions for progressing to later stages in the continuums. For example, in the treatment continuum, testing HIV seropositive is a necessary condition for receiving ART medication, while ART medication adherence is a necessary condition to achieving HIV viral suppression. The steps in this causal pathway contain “necessary” condition causes but these may not be “sufficient” causes to move a person to the next stage. Programs to link a person who has tested HIV seropositive to HIV treatment and programs to provide adherence support for individuals receiving ART may also be needed to move a person from a “necessary” causal condition to a “sufficient” causal condition.

In the prevention continuum, the causal pathways may be more complex, as there may be multiple pathways between the stages. For example, participating in a syringe service program is neither a necessary nor sufficient condition for having good supplies of sterile injection equipment. Nevertheless, the syringe exchange data clearly demonstrates that, at the PWID population level, implementing large scale syringe exchange programs does cause substantial increases in the numbers of PWID who have good supplies of sterile injection equipment [35] and thus can reduce syringe sharing. Participating in medication-assisted treatment (MAT) for opioid use disorders can be both a necessary and sufficient condition for many PWID to cease injecting drug use [36], which would necessarily cause them to cease sharing injection equipment, and thus avoid becoming infected with HIV through sharing syringes and injecting paraphernalia. Because not all persons receiving MAT cease injecting completely, however, that additional prevention interventions, such as large-scale NSP programs may be needed in order to reduce syringe sharing to acceptable levels.

By omitting information on deaths or the role of imprisonment, we may have inadvertently over-estimated coverage with prevention and care interventions among PWID. By omitting PWID who have stopped injecting drugs (on MAT) our model under-estimates coverage with prevention and care interventions. While the cross-sectional continuum is widely used for care continuum assessment, a longitudinal continuum, which incorporates loss to follow-up and mortality, might provide further insights about the performance of programmes [37]. For PWID, who are a hard to reach population, RDS is efficient for sampling. Yet, RDS samples may suffer from biases that we cannot detect. Self-reported data regarding awareness, uptake of services, adherence, and viral load counts (in 2016) is prone to recall and social desirability biases (that most likely would lead to over-estimates of coverage with prevention and care interventions).

Limitations also exist for our PCC steps and the indicators selected. Ignoring sexual transmission and related condom use is a limitation. For the PCC case study, we prioritized parenteral transmission as the primary mode of HIV transmission among PWID. For example, we rely on the last 6 months use of NSP for reporting current use, which might not be the ideal measure for consistency. However, 2013 data for the last four weeks and the last 6 months of NSP use were 72% and 75%, respectively, suggesting consistent use of NSP over this time-period. We had no data on further upstream indicators related to primary prevention, such as awareness of disease transmission via the sharing of used needles, but data from Estonia documents a high knowledge of HIV transmission modes among current PWID [14, 38].

Finally, the effectiveness estimates used to quantify the impact of interventions are uncertain, especially for NSP and ART. Specifically, for ART, strong evidence exists only for it decreasing sexual HIV transmission, which we assumed would also apply to parenteral HIV transmission. We also assume that the impact of each intervention acts independently, when in reality participation and individual effectiveness of interventions may vary by setting and mode of intervention and could even be driven by confounding factors such as personal motivation. Although better data is needed, these are the best estimates for the efficacy of these interventions and allow us to make insights on the likely degree to which their scale-up has decreased HIV risk.

However, we believe that these methodological limitations do not markedly impact our conclusions. The primary purpose of this work was to introduce a conceptual framework of the PCC. This demonstration study using available data was directed towards identifying if and how the framework could be implemented to enhance understanding, and ultimately inform the structuring and prioritizing of HIV care and prevention programs.

Individuals may progress through multiple care continua which may be inter-related to varying degrees. The consolidated continuum approach could be used for other diseases, such as hepatitis B and C, as it would be useful to assess the risk of infection and reinfection (e.g. among PWID in various countries where the treatment continuum may be similar but where varying levels of prevention will affect the risk of reinfection). The PCC is also dynamic; outcome measures might require modification if new treatment modalities are introduced, such as with hepatitis C, viral suppression shifts to cure [39]. The application of interventions may shift to a preventive stance, as with PrEP [40] or as new co-interventions become available.

### Conclusion

Developing a joint model for treatment/care and prevention continua emphasizes the bi-directional relationships between prevention and care. Stratified by the risk of acquiring or transmitting infection our analysis gives much needed information on how these subsets of population combine to produce an overall level of protection. Truly effective primary prevention implemented at the public health scale establishes an inviting pathway for diagnostic testing and linkage to care, thereby reducing infectious disease incidence as well as the disease burden for society. Expanding the care continuum to incorporate prevention can: 1) provide a theoretical framework that can inform future research in the field of HIV and other related diseases, 2) inform the development, implementation, evaluation and revision, and more efficient use of resources of prevention and care services in real world practice.

## Supporting information

S1 Data (XLSX)
